# Supplementary material for: Evaluating conflict-of-interest governance among Canadian medical societies using a novel assessment tool: A cross-sectional study
Source: PLoS One. 2026 May 14;21(5):e0348311. doi: 10.1371/journal.pone.0348311 (PMC13175343; doi:10.1371/journal.pone.0348311)
Supplement: S2 File — (DOCX) [file pone.0348311.s002.docx]

**Supplemental File 2**

| **Domain** | **Item** | **Rating scale** | **Scoring system** |
| --- | --- | --- | --- |
| Clinical practice guidelines | Prior to selection of the Guideline Development Group (GDG), individuals being considered for membership should declare all current and planned interests and activities potentially resulting in COI with development group activity | Required/Conditional/ Not required | 0, 1, 2 |
|  | Members of the GDG must divest themselves of financial investments they or their family members have in entities whose interests could be affected by CPG recommendations | Required/Conditional/ Not required | 0, 1, 2 |
|  | Members of the GDG must not participate in promotional activities, educational activities, speakers’ bureaus, advisory boards or boards of directors of entities whose interests could be affected by CPG recommendations | Required/Conditional/ Not required | 0, 1, 2 |
|  | Members with COIs must represent not more than a minority of the GDG | Required/Conditional/ Not required | 0, 1, 2 |
|  | The chair or co-chairs must not have any COI | Required/Conditional/ Not required | 0, 1, 2 |
|  | Sanctions for breaches of COI policy | Required/Conditional/ Not required | 0, 1, 2 |
|  | Industry funding of guidelines prohibited | Required/Conditional/ Not required | 0, 1, 2 |
|  | Disclosure of industry funding in guideline | Required/Conditional/ Not required | 0, 1, 2 |
|  | No industry input into topic of guideline | Required/Conditional/ Not required | 0, 1, 2 |
| External funding to society | Company funding | Not allowed/Conditional/ Allowed | 0, 1, 2 |
|  | Disclosure of exact amount received | Required/Partially disclosed (e.g., disclosed as a range or as a category)/ Not required | 0, 1, 2 |
|  | Name of company giving funding | Required/Conditional/ Not required | 0, 1, 2 |
| COI of society leadership including board | Current COI with industry/company allowed | Not allowed/Conditional/ Allowed | 0, 1, 2 |
|  | Current COI with industry/company | Disclosed publicly/Conditional/ Not disclosed publicly | 0, 1, 2 |
|  | Previous COI with industry/company allowed | Not allowed/Conditional/ Allowed | 0, 1, 2 |
|  | Previous COI with industry/company | Disclosed publicly/Conditional/ Not disclosed publicly | 0, 1, 2 |
| COI of society staff | Current COI with industry/company allowed | Not allowed/Conditional/ Allowed | 0, 1, 2 |
|  | Current COI with industry/company | Disclosed publicly/Conditional/ Not disclosed publicly | 0, 1, 2 |
|  | Previous COI with industry/company allowed | Not allowed/Conditional/ Allowed | 0, 1, 2 |
|  | Previous COI with industry/company | Disclosed publicly/Conditional/ Not disclosed | 0, 1, 2 |
| Continuing Medical Education/Continuing Professional Development | Independent development of topic by society | Required/Conditional/ Not required | 0, 1, 2 |
|  | Independent development of content by society | Required/Conditional/ Not required | 0, 1, 2 |
|  | Adherence to Royal College of Physicians and Surgeons of Canada, National Standard for Support of Accredited CPD Activities | Required/Conditional/ Not required | 0, 1, 2 |
|  | Company booths | Not allowed/Conditional/ Allowed | 0, 1, 2 |
|  | Company funding for meeting | Not allowed/For particular event at meeting/ No restrictions | 0, 1, 2 |
|  | Disclosure of funding | Exact amount disclosed/Amount put into category (e.g., platinum, gold *or* dollar range)/ No disclosure of amount or category | 0, 1, 2 |
|  | Unaccredited CME/CPD activities organized by companies during society sponsored CME/CPD activity | Not allowed/Conditional/ Allowed | 0, 1, 2 |
|  | Unaccredited CME/CPD activities clearly identified as such | Required/Conditional/ Not required | 0, 1, 2 |
|  | Key society leadership participation in unaccredited CME/CPD activities organized by companies | Not allowed/Conditional/ Allowed | 0, 1, 2 |
|  | Speakers’ declaration of COI | Required/Conditional/ Not required | 0, 1, 2 |
|  | Speakers’ use of company produced slides | Not allowed/Conditional/ Allowed | 0, 1, 2 |
| Annual general meeting | Company booths | Not allowed/Conditional/ Allowed | 0, 1, 2 |
|  | Funding for meeting | Not allowed/For particular event at meeting/ No restrictions | 0, 1, 2 |
|  | Disclosure of funding | Exact amount disclosed/Amount put into category (e.g., platinum, gold *or* dollar range)/No disclosure of amount or category | 0, 1, 2 |
| Research funding | Society determines nature of the project | Required/Conditional/ Not required | 0, 1, 2 |
|  | Project is externally peer reviewed | Required/Conditional/ Not required | 0, 1, 2 |
|  | Society determines study design | Required/Conditional/ Not required | 0, 1, 2 |
|  | Society solely responsible for data analysis | Required/Conditional/ Not required | 0, 1, 2 |
|  | Society determines publication | Required/Conditional/ Not required | 0, 1, 2 |
| Policy on relationship with industry | Publicly available | Required/Conditional/ Not required | 0, 1, 2 |
|  | Periodic review of policy | Required/Conditional/ Not required | 0, 1, 2 |
|  | Sanctions for breach of policy | Required/Conditional/ Not required | 0, 1, 2 |
|  | Existence of committee responsible for ensuring compliance | Required/Conditional/ Not required | 0, 1, 2 |
|  | Use of society name and logo by company | Not allowed/Conditional/ Allowed | 0, 1, 2 |
|  | Contact with society membership by company | Not allowed/Allowed with restrictions/ Allowed | 0, 1, 2 |
|  | Endorsement of product(s) sold by company or policies/initiatives advanced by company | Not allowed/Conditional/Not mentioned/Allowed | 0, 1, 2 |
|  | Hyperlink from donor logo on society website to donor website | Not allowed/Conditional/ Allowed | 0, 1, 2 |
| Society journal | Editorial independence | Required/Conditional/ Not required | 0, 1, 2 |
|  | Authors required to declare COI | Required/Conditional/ Not required | 0, 1, 2 |
|  | Editors and reviewers required to disclose COI | Required/Conditional/ Not required | 0, 1, 2 |
|  | Journal prohibits ghost authorship or ghost management | Required/Conditional/ Not required | 0, 1, 2 |
