# Supplementary material for: Evaluating conflict-of-interest governance among Canadian medical societies using a novel assessment tool: A cross-sectional study
Source: PLoS One. 2026 May 14;21(5):e0348311. doi: 10.1371/journal.pone.0348311 (PMC13175343; doi:10.1371/journal.pone.0348311)
Supplement: Attachment — Submitted filename: Response to Reviewers.docx [file pone.0348311.s004.docx]

Response to Reviewers

Reviewer #1:

| **Reviewer #1 comments** | **Authors’ response** |
| --- | --- |
| The manuscript is well written and organized, and the data availability and reporting standards meet PLOS ONE requirements. I have no major concerns except from some minor issues | Thank you for your positive feedback upon your reading of our manuscript. |
| Typo/wording: “Policy presense” in the Results section should be “Policy presence.” | Thank you for spotting this typo, we have now corrected the spelling. |
| Consistency and clarity: Consider defining early what counts as a “policy item” vs. a “domain,” then keep the wording consistent throughout. | Thank you for flagging that this distinction was not made clear from the outset. We have now revised the paragraph in the methods section under ‘Tool to analyze policies’ to clarify how these terms are interpreted. Specifically, we now say “The resulting tool consisted of discrete *policy items* (i.e. specific policy provisions or rules) grouped into broader thematic *domains* (i.e. categories of related policy items addressing a common policy area.” |

| **Reviewer #2 comments** | **Authors’ response** |
| --- | --- |
| The manuscript presents a novel, systematic evaluation of conflict-of-interest (COI) policies among Canadian medical societies. The topic is of high relevance to medical ethics, professional self-regulation, and public trust. The study is well-written, well-structured, the methodology is clearly described, and the findings are stark and impactful, clearly demonstrating a major gap in transparent COI governance. However, several major methodological and interpretive issues need to be addressed to strengthen the validity of the conclusions and the utility of the tool, these include | We are very grateful for your time in engaging with our manuscript. We have enjoyed engaging with your suggestions and feel the manuscript has been strengthened as a result. |
| *Major comments* |  |
| 1. The core of this study is the novel 51-item tool. While the expert review process is noted, the manuscript does not thoroughly address the tool's construct validity in terms of system scoring, Item and Domain Selection, and weighing. | Thank you for your suggestion to expand our discussion around the tool’s construct validity. In response, we have expanded the Methods section to clarify the conceptual and methodological foundations of the assessment tool, in particular please see the revised “Tool to analyze policies” subsection.  Specifically, we now describe (1) the literature-informed process used to identify and synthesise policy items from prior institutional COI governance frameworks; (2) the rationale for domain structure based on areas where conflicts of interest have been shown to influence medical society activities – for each domain we now include a reference; (3) the iterative expert review process involving 10 researchers with extensive experience in COI policy development and analysis; and (4) the rationale for the scoring system and equal weighting of items to ensure transparency and avoid subjective prioritisation. We also note that this development approach is consistent with established methods used in prior institutional COI policy assessment tools, specifically in academic medical centres. |
| 2. It is crucial to have a robust justification for the decision and its ramifications, as limiting the search to public websites represents a major methodological choice. | We thank you for highlighting the need to more clearly justify this methodological decision. We have revised the Methods section to clarify that restricting the search to publicly accessible policies was intentional and to explain the implications of this choice. The revised text now states: “Policies were identified through a systematic search of publicly accessible sections of each society’s website.” We further clarify the rationale for this approach: “We focused on publicly available policies because these represent the standards communicated to external stakeholders and therefore constitute the most transparent and publicly accountable form of organizational governance.”  We also explain the implications for reproducibility and data collection, noting that “Restricting the search to publicly accessible materials also ensured methodological reproducibility, as access to password-protected or internal documents varies across organizations and investigators.” Finally, we clarify that societies were not contacted and specify the scope of the analysis: “We did not contact medical societies to request additional policies because our objective was to evaluate policies accessible to any interested party. Consequently, our analysis reflects the policies that societies make publicly available rather than any internal policies that may exist.” |
| 3. Tables 3, 4, and 6 are dense and somewhat confusing. Table 3's "Depth of coverage" columns are hard to parse. Consider simplifying. For Table 6, the "% of Max" column is useful but requires careful reading. Ensure the caption/footnote clearly explains that a lower percentage indicates a more restrictive overall policy stance. | Thank you for your reflections on the Tables, this is very helpful and we have made some amendments as a result.  To address the confusing presentation of Table 3 (now Table 4), we have divided it into two separate tables. Previously, it was combining breadth and depth which we agree made it confusing for the reader. Splitting the Table into two also has the added benefit of better aligning with the text, as we have a section for breadth and a section for depth.  We have added an additional sentence to the footnote for Table 6 (now Table 7) to clarify how readers can interpret the scores – “**Percentage of maximum represents the absolute score as a percentage of the maximum (most permissive) possible score if all items were scored as 2. Lower percentages indicate more restrictive overall policy positions, whereas higher percentages indicate more permissive policies.”* |
| 4. The low "depth" scores are highlighted. This could be interpreted not only as a policy weakness but also that the tool is very comprehensive. This dual interpretation should be acknowledged. | We appreciate this point and have inserted an additional sentence to reflect the dual interpretation, as you suggested. We now state: “Even within policies, depth of coverage was modest: on average, societies addressed fewer than one-third of the relevant items within each domain. While this could be interpreted as a policy weakness, it may also reflect the comprehensiveness of our tool.” |
| 5. The discussion suggests policies are "reactive" and "compliance-driven." While plausible, this is still a speculative from a cross-sectional study. It is thus strongly suggested to refine informal phrasing (such as “implying” or “indicating”) to underscore the relational elements within your observations. | Thank you for highlighting this. We appreciate and agree there is a need to avoid speculative interpretations and causal language as this is not supported by our research design.  We have revised with discussion section to use more cautious, relational phrasing that emphasises observed policy characteristics rather than institutional intent or motivations. Specifically:   - We replaced stronger interpretative language with observational phrases (e.g. "more frequently observed”, “more frequently included”, “consistent with”, “may suggest”, and “may have implications”). - We reframed statements about policy orientation to focus on structural patterns (e.g. “consistent with COI governance being structured around meeting external requirements”) rather than implying institutional motives - We clarified interpretative statements by introducing hedging language (e.g. “may”, “suggest”, “comparatively”). - We revised statements about potential impacts to emphasise implications rather than direct causal effects.   We retained one instance of the term “reactive”, but softened the phrasing to: “This pattern may suggest a comparatively reactive rather than proactive approach,” which we believe conveys a cautious interpretation of observed policy features rather than a claim about organisational intent.”  We feel these revisions ensure that our discussion remains proportionate to the study design while preserving the policy-relevant insights of our findings. |
| *Minor comments* |  |
| 1. In the abstract, the authors wrote "Systematically searched their websites". Could be slightly more specific (e.g., "publicly accessible sections of their websites"). | Thank you for this helpful suggestion. We have clarified the scope of our search by revising the sentence to specify that only publicly accessible website content was reviewed. The sentence now reads: “We identified 68 Canadian medical societies meeting our inclusion criteria and systematically searched the publicly accessible sections of their websites for COI policies.” |
| 2. Line 38: Typo: "a 38 nd staff COI" -> "and staff COI". | Thank you for pointing this out. This typo did not appear on our original Word copy, but may have occurred during the submission process. We have ensured that it does not appear in the revised version. |
| 3. Line 288: "The analysis of the policies does not apply to policies of excluded Canadian organizations..." This sentence is awkward. Rephrase for clarity. | Thank you for pointing this out to us. We have now rephrased this sentence so it reads: “Our findings do not extend to organizations excluded under our eligibility criteria (e.g. regulatory colleges, accrediting bodies, broad physician associations, or multidisciplinary organizations) or to medical societies in other countries.” |
| 4. In Discussion section (Subtle: International Comparison): The comparison with the Australian study (Kerridge et al. 2005) is excellent as it contrasts your finding on speaker declarations. Emphasize this point of difference more. | Thank you for identifying this as an important point of contrast between our findings and other contexts. We have sharpened the phrasing to draw more attention to this fact by adding an additional sentence:  “In Australia, Kerridge et al. (19) reported that 20 of 29 responding organizations (from 63 contacted) had policies or guidelines governing industry relationships. However, these policies appeared limited in scope: 62% of organizations did not require speakers to declare COIs at educational meetings. This contrasts with our findings, where speaker declaration requirements was the most frequently addressed policy item across all items in our study. The increase in the percent of policies requiring speaker disclosure may reflect an expanding recognition of the influence of COI” |
